# Where to Restore Ecological Connectivity? Detecting Barriers and Quantifying Restoration Benefits

**DOI:** 10.1371/journal.pone.0052604

**Published:** 2012-12-27

**Authors:** Brad H. McRae, Sonia A. Hall, Paul Beier, David M. Theobald

**Affiliations:** 1 The Nature Conservancy, North America Region, Seattle, Washington, United States of America; 2 The Nature Conservancy, Washington Chapter, Wenatchee, Washington, United States of America; 3 School of Forestry and Merriam-Powell Center for Environmental Research, Northern Arizona University, Flagstaff, Arizona, United States of America; 4 National Park Service, Inventory and Monitoring Division, Fort Collins, Colorado, United States of America; University of California, Berkeley, United States of America

## Abstract

Landscape connectivity is crucial for many ecological processes, including dispersal, gene flow, demographic rescue, and movement in response to climate change. As a result, governmental and non-governmental organizations are focusing efforts to map and conserve areas that facilitate movement to maintain population connectivity and promote climate adaptation. In contrast, little focus has been placed on identifying barriers—landscape features which impede movement between ecologically important areas—where restoration could most improve connectivity. Yet knowing where barriers most strongly reduce connectivity can complement traditional analyses aimed at mapping best movement routes. We introduce a novel method to detect important barriers and provide example applications. Our method uses GIS neighborhood analyses in conjunction with effective distance analyses to detect barriers that, if removed, would significantly improve connectivity. Applicable in least-cost, circuit-theoretic, and simulation modeling frameworks, the method detects both complete (impermeable) barriers and those that impede but do not completely block movement. Barrier mapping complements corridor mapping by broadening the range of connectivity conservation alternatives available to practitioners. The method can help practitioners move beyond maintaining currently important areas to restoring and enhancing connectivity through active barrier removal. It can inform decisions on trade-offs between restoration and protection; for example, purchasing an intact corridor may be substantially more costly than restoring a barrier that blocks an alternative corridor. And it extends the concept of centrality to barriers, highlighting areas that most diminish connectivity across broad networks. Identifying which modeled barriers have the greatest impact can also help prioritize error checking of land cover data and collection of field data to improve connectivity maps. Barrier detection provides a different way to view the landscape, broadening thinking about connectivity and fragmentation while increasing conservation options.

## Introduction

Landscape connectivity, or “the degree to which the landscape facilitates or impedes movement among resource patches” [1], is crucial for many ecological and evolutionary processes, including dispersal, gene flow, demographic rescue, and movement in response to climate change [2]–[7]. Many research and conservation planning efforts have focused on mapping areas important for connectivity using GIS models (e.g., [8]–[12]). The results of these analyses are guiding investments by governmental and non-governmental organizations to promote ecological connectivity across large areas. In the USA and Canada, for example, numerous broad-scale conservation efforts such as the U.S. Department of Interior Landscape Conservation Cooperatives, the Western Governors' Association's Initiative on Wildlife Corridors and Crucial Habitat, and the Yellowstone to Yukon Conservation Initiative are working to integrate and coordinate connectivity conservation actions spanning millions of acres and crossing many political and ecoregional boundaries.

Conservation practitioners employ two primary strategies to promote connectivity. The first focuses on conserving areas that *facilitate* movement; the second focuses on restoring connectivity across areas that *impede* movement (e.g., by removing a fence or building a wildlife-friendly highway underpass). Most connectivity analyses have focused on the former strategy by modeling and mapping areas important for movement under present landscape conditions. A wide array of tools have been developed for this purpose: least-cost corridor modeling [8], [13], [14], circuit theory [15], individual-based movement models (e.g., [16]–[18]), graph theory [19], [20], and centrality analyses (e.g., [21], [22]) have all been used to identify areas important for movement of plants and animals. Outputs from such models are now being used as inputs to reserve selection algorithms (e.g., [23]) to optimize actions to conserve connectivity.

In contrast, there has been little effort by conservation scientists towards identifying candidate areas for the second strategy: that is detecting restoration opportunities by mapping barriers that strongly reduce movement potential. We define a barrier as a landscape feature that impedes movement between ecologically important areas, the removal of which would increase the potential for movements between those areas. Here we are concerned with movements important for access to resources, demographic rescue, gene flow, range shifts, and other ecological and evolutionary processes. In this context, barriers are distinguished from features that are impermeable but not situated such that they block biologically relevant movement routes. Barriers are thus the inverse of corridors, which delineate pathways facilitating movement. Barriers can either be complete (impermeable) or partial (e.g., land cover types that hinder movement relative to ideal conditions, but may still provide some connectivity value). Barriers may be human-made (e.g., roads, fences, or urban areas) or natural (rivers or canyons); they may be linear (e.g., highways) or span large areas (agricultural fields). As with traditional connectivity concepts [1], [24], what constitutes a barrier, the impact it has, and whether it reduces connectivity through behavioral inhibition, increased mortality, or other means will differ among species.

Detecting barriers to movement would complement traditional connectivity analyses in several important ways. First, some barriers may be restorable. Knowing where barriers have the greatest impact would help practitioners decide where and how to invest scarce conservation resources to conserve and enhance connectivity. For example, it may be cheaper to restore a barrier that blocks a movement corridor through public land than to establish permanent protection of a functioning corridor that runs through private land [25]. Quantifying such trade-offs would be necessary to integrate connectivity restoration into systematic conservation planning analyses aimed at optimizing conservation investments [26]–[28], but tools to incorporate connectivity conservation and/or restoration into such efforts remain rare [29]–[31]. Second, consider that corridor modeling often produces corridors that may not be good enough to realistically support movement [32]. Barrier detection analysis could reveal such cases, allowing practitioners to ‘triage’ a landscape, focusing efforts on more viable movement routes. Finally, surprising results in a barrier analysis could alert analysts to situations in which poor land cover data or incorrect model parameterization may be causing spurious results.

In this paper, we introduce a new method to identify barriers and rank them by their impact on connectivity. Our method complements existing connectivity modeling approaches, is applicable in least-cost and other connectivity modeling frameworks, and can be extended to centrality analyses. The method can be readily applied across large landscapes, efficiently analyzing barriers among many locations and at different scales corresponding to different sizes of barriers and types of restoration activities. It also quantifies the extent to which restoration can be expected to improve connectivity. We provide example applications of the method, showing that the potential for connectivity conservation is not constrained to narrow corridors, but includes options spanning much more of the landscape when restoration options are considered. We also discuss how our approach can facilitate sensitivity analyses, data quality screening, and prioritization of areas for error checking of GIS base data.

## Method for Detecting Barriers and Restoration Opportunities

Our method identifies areas that most reduce connectivity between two locations on a landscape. Making these areas permeable to movement would therefore most increase connectivity between the locations. Thus, these are areas that practitioners should consider when implementing restoration to promote connectivity.

To illustrate the method, we use a least-cost corridor modeling framework [13], [14], [32], which is commonly used to map and prioritize areas important for connectivity conservation (e.g., [8]–[12]). However, our approach could also be used with other modeling frameworks capable of producing measures of effective distance, such as circuit theory and individual-based movement models (see Discussion).

**Figure 1:**
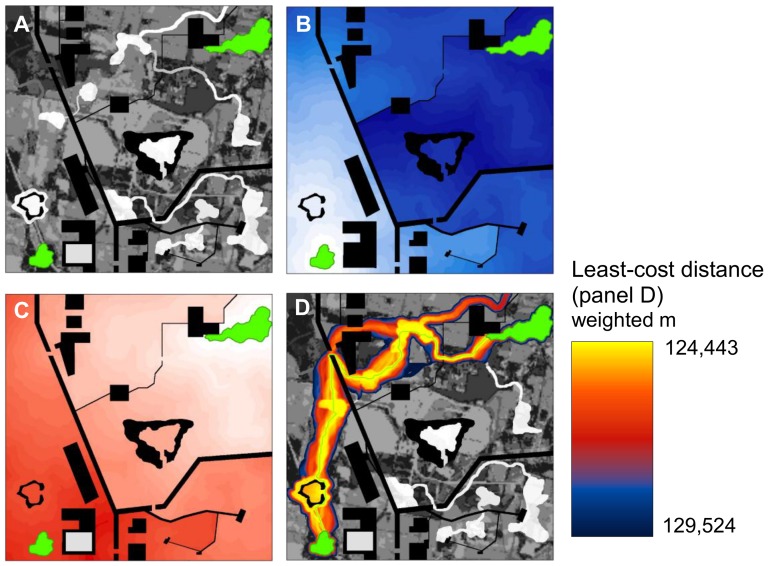
Cost-weighted distance modeling. (A) Example 3 km×3 km landscape with a pixel size of 3 m (from [15]). Two habitat patches (green) are embedded in a matrix of land cover types with differing resistance to movement. Resistances range from 1 (white) to 100 (dark grey); complete barriers with infinite resistance (e.g., linear features representing roads and highways) are shown in black. (B) Cost-weighted distance (*CWD*) from leftmost patch, with darker shades representing higher cumulative resistance from the patch. (C) *CWD* from rightmost patch, with darker shades representing higher cumulative resistance from the patch. (D) Modeled least-cost corridor produced by adding *CWD* surfaces shown in panels B and C (best 20% of study area shown). The least-costly path (traced in green) has a cumulative least-cost distance (*LCD*) of 124,443 weighted meters.

As with least-cost corridor models, input data include locations to be connected (hereafter, “patches”) and a raster resistance surface (Figure 1A). The former may consist of points or polygons, and typically represent natural landscape blocks, protected areas, or core habitat for a particular species or species guild [33]. The resistance surface represents the difficulty, energetic cost, or mortality risk associated with movement through each pixel (see [34] for a review of resistance surface development).

Least-cost methods calculate the cost-weighted distance (*CWD*) of all pixels to a source location, creating a raster of *CWD* values (Figures 1B and 1C). Adding together *CWD* rasters from two locations produces a corridor (Figure 1D), showing the pathways with the lowest cumulative movement cost between the locations [14]. The minimum value of the corridor raster is the least-cost distance (*LCD*); this represents the cumulative resistance encountered moving along the optimal path from one location to the other, and is a common measure of isolation in spatial ecology (e.g., [35], [36]), landscape genetics (e.g., [37], [38]), and related fields.

Our method is based on this simple assumption: if a certain area (the size is defined by the user) is restored such that the resistance across it is reduced, then the *LCD* of the best route connecting the patches through the restoration area will also be reduced. Systematically quantifying the potential reduction across a landscape will allow us to detect those areas where restoration would lead to the greatest reduction in least-cost distance.

**Figure 2:**
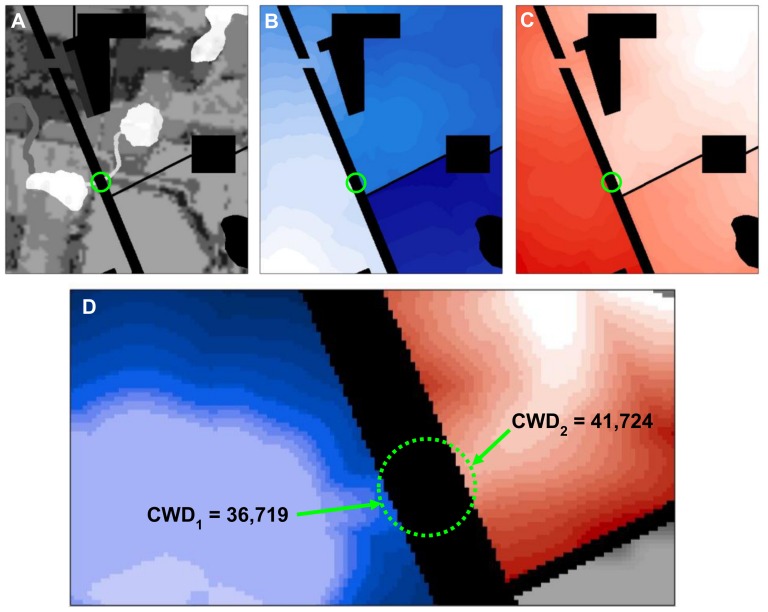
Detail of resistance and *CWD* surfaces with circular moving window. For a window with a diameter of 60 m (20 pixels) centered on the barrier, the arrows show the pixels in the window that have the lowest *CWD* to each patch (values shown are in weighted meters). Because the lowest *CWD* values from each patch will always be found on the edge of a moving window, only pixels on the perimeter need to be examined, increasing processing efficiency.

The method begins with *CWD* calculations from two patches (Figures 1B and 1C). However, rather than adding the two *CWD* surfaces together to produce a corridor, we instead calculate the minimum value of each *CWD* surface within a localized area around each pixel location (e.g., within a 500 m radius). We then add the minimum values from both *CWD* surfaces to calculate the cumulative resistance that would be incurred moving between the patches and through the focal pixel assuming the area within the search window is restored:
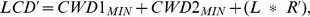
(1)where *LCD′* is the least-cost distance of the best path between the patches passing through the focal pixel after barrier removal, *CWDX_MIN_* is the minimum *CWD* value from patch *X* within the search window, *L* is the length of the longest axis of the search window, and *R′* is the resistance value of the feature replacing (or cutting through) the barrier. We use a circular moving window to illustrate the method (Figure 2), but consider alternative search window shapes in the Discussion. Note that the longest axis of a circle is its diameter.

For each pixel, this formula yields the cost of the best corridor that would pass through that pixel if the resistance of a strip of land crossing the search window were changed to *R′*. Including *R′* and the search window length accounts for the cost of moving across the search window, assuming restoration or removal of the intervening barrier.

If *LCD′* is less than *LCD*, then restoration across the moving window (e.g., the circle in Figure 2) would reduce effective distance and increase connectivity between the two patches. When this is the case, a simple metric of connectivity improvement that would result from restoration across the moving window is:
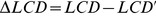
(2)Dividing *ΔLCD* by the search diameter gives the connectivity benefit per unit distance restored; dividing *ΔLCD* by *LCD* gives the proportional improvement relative to unrestored effective distance.

**Figure 3:**
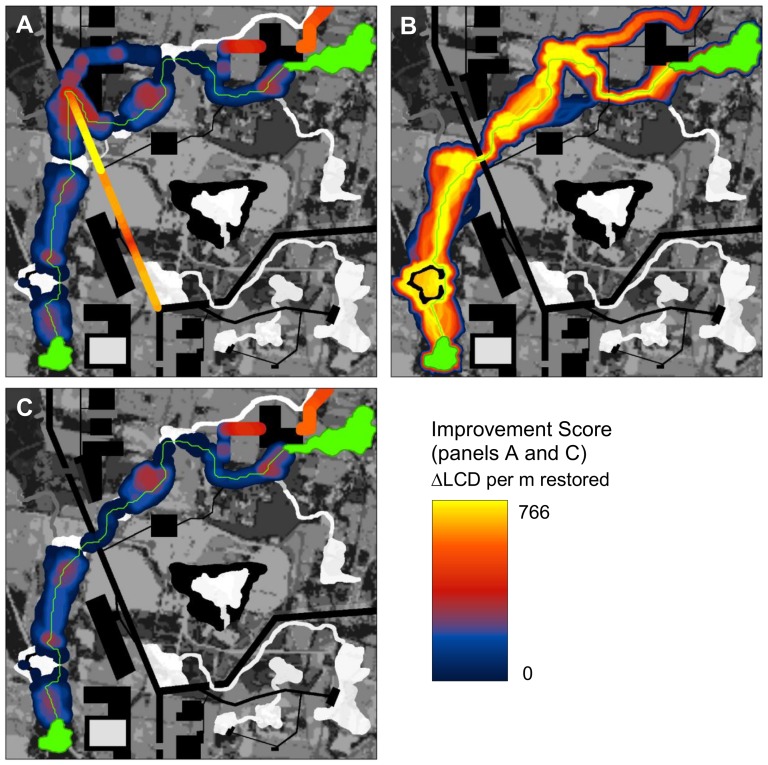
Barrier analysis of landscape. (A) Improvement scores (*ΔLCD*) for a 60 m search diameter using an enhanced version of Linkage Mapper software ([40]). Only positive values (indicating barriers whose removal would reduce isolation) are shown. To facilitate visualization of the barriers, scores were mapped so that they filled the search window (i.e. the maximum *ΔLCD* value within the search radius of each pixel is displayed). The greatest improvement potential was detected crossing the highway. Note that a natural corridor is bisected by the highway at the point with the highest improvement potential (see detail in Figure 2). (B) Creating a new gap in the barrier where restoration potential is highest re-routes the modeled least-cost corridor and greatly reduces resistance between the patches (*LCD* = 78,503 weighted meters compared with an *LCD* of 124,443 pre-restoration). Best 20% of study area shown. (C) Barrier detection at 60 m search diameter after restoration.

To illustrate the method, we first apply it to the relatively simple landscape described in Figure 1 using a search window with a diameter of 60 m (20 pixels at 3 m resolution; Figure 2). The search window size is chosen to match the size of the barrier that one is interested in detecting: a diameter of 20 pixels will fully incorporate effects of barriers up to 20 pixels across. We assign a resistance of 1 to optimal movement habitat, so that the cumulative cost of movement is identical to the Euclidean distance traversed when no barriers are encountered. For the circular window centered on the highway in Figure 2, the lowest *CWD* values from the left and right patches are 36,719 and 41,724 weighted meters, respectively. Summing these values and adding 60 (the cost of crossing the circle if it were restored to optimal movement habitat with a resistance of 1), gives the least-cost distance of the path crossing through the restored area (36,719+41,724+60 = 78,503 weighted meters). Since this is considerably lower than the least-cost distance between the patches without restoration (124,443 weighted meters), this location is a potent barrier, and the center pixel is assigned an improvement value of 45,940 weighted meters. This is repeated for every pixel on the landscape using standard GIS neighborhood analyses, resulting in a raster surface of improvement scores (Figure 3A).

The removal of the barrier where the improvement score is maximal – for example, by constructing a wildlife crossing structure – would re-route the best movement path (Figure 3B) and lower the effective distance between the two patches by 37% (45,940/124,443). Once that improvement is carried out, a second barrier analysis with the altered landscape conditions suggests that additional restorations along the highway will not further reduce the *LCD* at this point (Figure 3C). The next priority would be a road crossing in the upper right of the panel (dark orange in Figure 3C), connecting the rightmost patch to high-quality movement habitat above the road. The method is computationally efficient enough that different restoration scenarios can be tested iteratively: a barrier analysis with a 20- pixel search diameter across a landscape with 1 million pixels takes less than 2 seconds using a 2.7 GHz notebook computer.

### Identifying barriers across scales and across large landscapes with multiple patches

**Figure 4:**
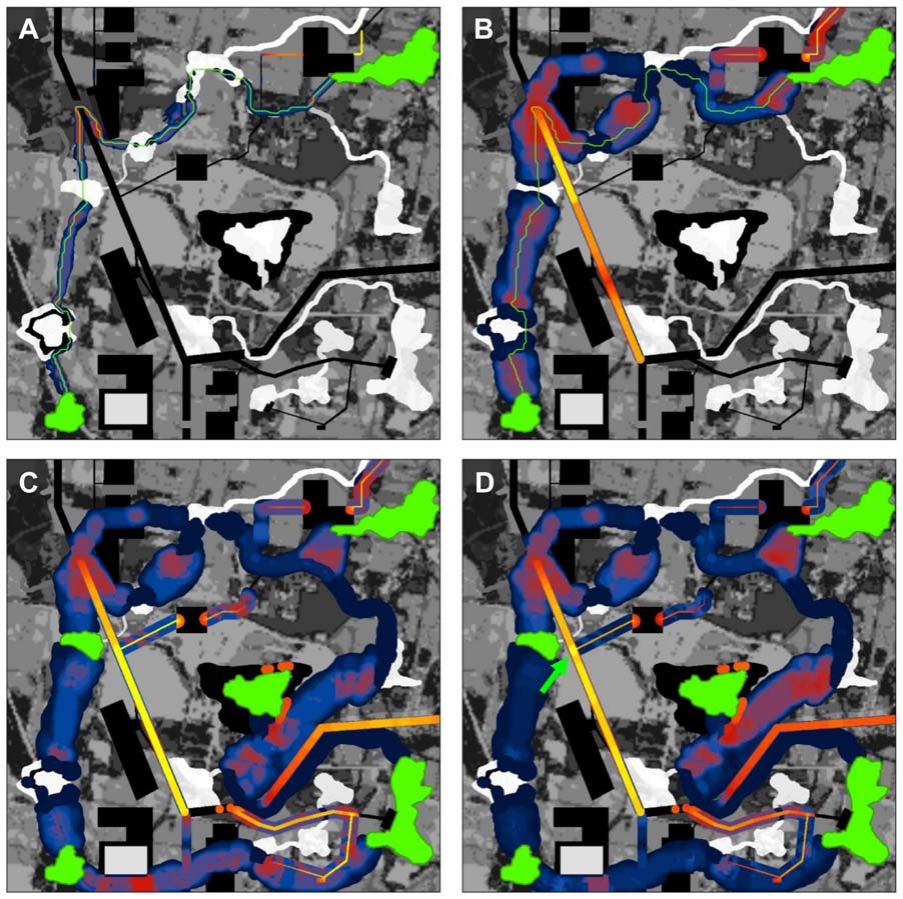
Barrier analyses integrating across multiple scales and patch pairs. (A) Results of barrier analysis with original patch pair at 12 m search diameter, which detects restoration opportunities equal to or less than 12 m across (e.g., local roads). (B) Maximum per-meter improvement value across 10 search window sizes (from 6 m to 60 m, with 6 m steps between search diameters). The map highlights where actions at different scales would have highest impact per meter restored. (C) Maximum per-meter improvement value across same window sizes and 5 patches, showing where greatest improvement could be achieved for any single pair of patches. (D) Sum of improvement scores among 5 patches (green). As in Panels A–C, the maximum per-meter improvement score was calculated for each patch pair at each scale. These were then summed across patch pairs to incorporate cumulative benefit for multiple patch pairs across multiple scales. The area scoring highest (bright yellow) had high improvement scores for multiple patch pairs; we interpret this area as having high ‘barrier centrality,’ i.e. being an important restoration opportunity for keeping the overall network connected. Note that the area occurs at a road intersection; if practical, placing a wildlife crossing structure here would re-route four corridors connecting the two leftmost patches to both the central and upper-right patch.

The method described above can be extended across scales and across networks of patches, and we explore a few approaches to accomplish this here. By modifying the search diameter, the method can detect barriers of different sizes (Figure 4). Windows the width of a highway will best highlight where highways act as barriers, as in Figures 2 and 3. Larger windows will best detect barriers like agricultural fields, or cases in which narrow barriers run parallel to one another. Summary maps showing barrier effects across search window sizes may be created by first dividing improvement scores by the window size to produce maps of barrier strength per unit width, and then taking the maximum pixel score across scales (Figure 4B). This puts results from different analysis scales in the same units, allowing them to be summarized in a single map. Alternative summary metrics are possible, and we address some of them in the Discussion.

To summarize across multiple sets of patch pairs, we have implemented a similar approach in which the maximum or sum of improvement scores across all patch pairs is assigned to a pixel. Taking the maximum of improvement scores shows the features that have the greatest effect for any patch pair (Figure 4C). Summing improvement scores highlights those barriers that isolate multiple pairs of patches from one another, extending the method to quantify barrier centrality (Figure 4D).

The methods described in this paper have been implemented in Barrier Mapper software [39], freely available as a new addition to the Linkage Mapper Toolkit for ArcGIS [40].

### Example application in a landscape undergoing active conservation planning

The Washington Wildlife Habitat Connectivity Working Group, a collaboration of land and resource management agencies, non-governmental organizations (NGOs), universities, and Washington treaty tribes, recently completed a connectivity analysis across the Columbia Plateau Ecoregion in Washington, Oregon, and Idaho, USA [41]. The Working Group focused on the Columbia Plateau because the ecoregion is home to a large portion of Washington's sensitive plant and animal species but is also highly fragmented by agriculture and other anthropogenic activities. The Group modeled corridors to connect habitat for 11 focal species and also to connect natural landscape blocks scoring highly on an index of landscape integrity (i.e., large areas with relatively low levels human modification). Products from the analysis are being used to inform conservation planning efforts by several state and federal agencies and NGOs. Many of the corridors identified by the analysis pass through human-dominated landscapes, where roads, agricultural fields, and other human uses likely still act as barriers to movement.

**Figure 5:**
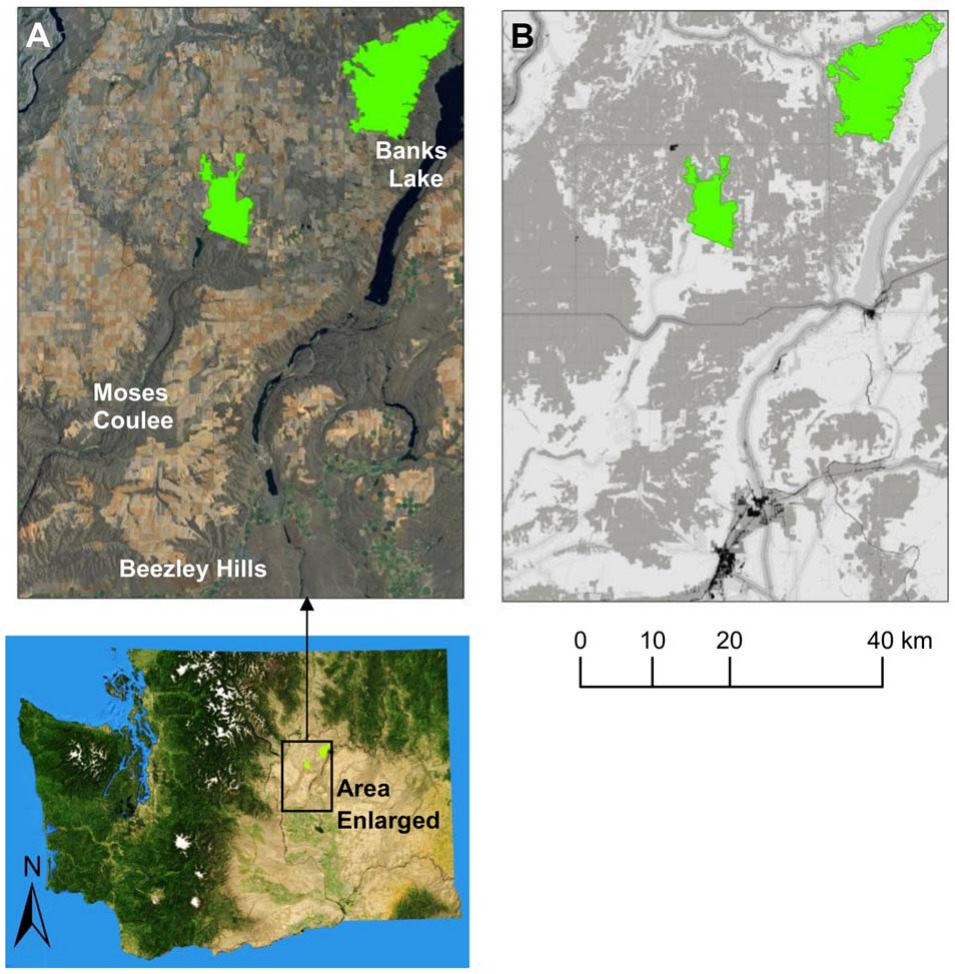
Corridor analysis in a landscape undergoing active conservation planning. (A) 60 km by 80 km study area in eastern Washington, USA, containing two natural landscape blocks to be connected (green). (B) Resistance map used to model corridors in a recent multi-partner connectivity analysis across the Columbia Plateau Ecoregion [41]; values range from 1 (white) to 1000 (black). Low resistance areas include native grassland and shrub-steppe, whereas high resistance areas include roads, developed areas, and agriculture.

We reanalyzed results for a corridor connecting two natural landscape blocks identified by the Working Group in Douglas County, Washington (Figure 5). We chose these blocks because they have been identified as important for many species of concern; for example, the blocks contain important habitat or corridors for 8 of 11 focal species analyzed by the Working Group. Moreover, both are occupied by greater sage-grouse (*Centrocercus urophasianus*, categorized as a Species of Greatest Conservation Need in Washington and a candidate for listing under the US Endangered Species Act), and both fall within a recovery area designated for the species by Washington State [42]. In addition, this landscape contains a complex mix of native systems and agricultural lands – the latter including both annual cropland and perennial vegetation cover – and includes roads, transmission lines, and other human-made features affecting animal movement [41].

To represent species with differing degrees of sensitivity to human modification, the Working Group used different resistance surfaces for landscape integrity analyses [41]. These surfaces all contained resistance values that increased with the degrees of human modification, differing only in the range of resistances assigned. Resistance scores of 1–100, 1–1000, and 1–10,000 were used for minimum, medium, and maximum sensitivity surfaces respectively (see [41] for details). We present results from a barrier analysis using the medium sensitivity resistance surface.

**Figure 6:**
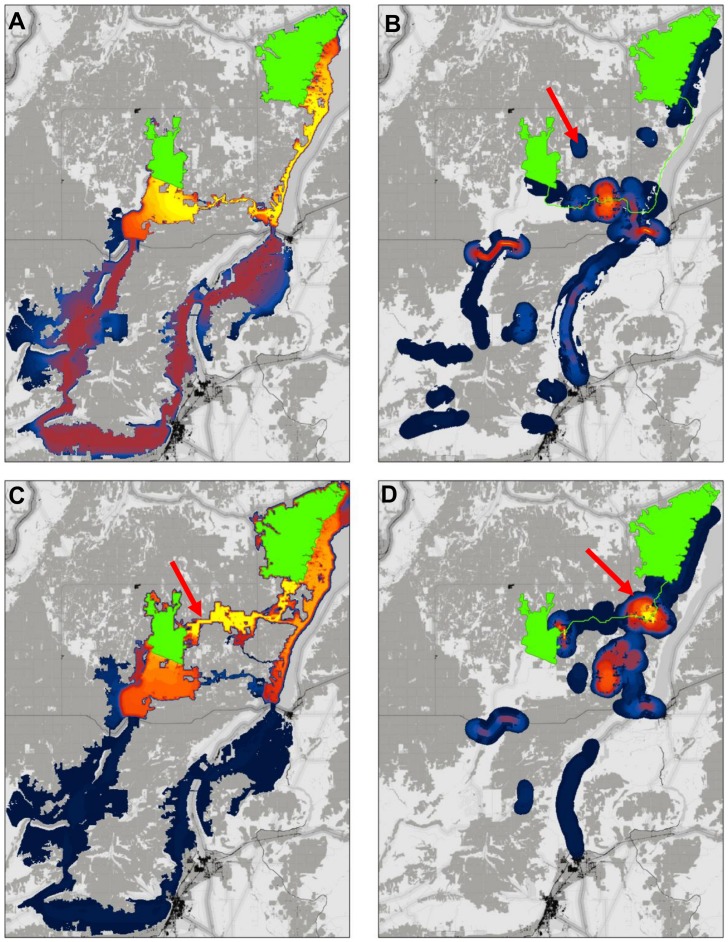
Reanalysis of connectivity modeling results using barrier detection algorithm. (A) Corridor connecting natural landscape blocks, showing least-cost movement routes. Best 20% of study area shown. (B) Barriers detected at diameters from 200 m to 2 km, with original least-cost path shown in green for reference. Mitigating barriers along the least-cost path (i.e., intersecting the green line) would improve the existing corridor without changing its location; mitigating barriers away from the path would re-route the best modeled corridor. (C) Restoring a 1 km^2^ (500 m×2 km) swath spanning the barrier indicated by the arrow establishes a new least-cost corridor to the North. (D) A barrier analysis incorporating the simulated restoration indicates opportunities to substantially improve the new corridor with additional restorations.

The modeled least-cost corridor connecting the patches dips south from the western patch, runs east to Banks Lake, and then north along a narrow strip of native vegetation and cliffs sitting between the lake and cropland (Figure 6A). A secondary and much longer corridor follows broad swaths of native vegetation through Moses Coulee and Beezley Hills to the south. A barrier analysis indicates numerous opportunities for improving the least-cost corridor, particularly within its east-west segment (Figure 6B). There are also opportunities outside of the main corridor, occurring along the longer route to the south and to the north as well (Figure 6B). Restoring any of these latter areas would re-route the modeled least-cost corridor, causing it to occur in a different location than it did in the unrestored landscape.

Restoration of any of several barriers identified to the south would improve connectivity as measured by *LCD* (Figure 6B); however, this would result in a much longer least-cost corridor. Restoration to the north has the potential to both improve *LCD* and shorten the distance traversed by the corridor. We simulated a restoration by changing a 1 km^2^ (500 m×2 km) swath of agricultural land (indicated by the arrow in Figure 6B) to a resistance of 1. We chose 2 km because the greatest improvement was detected at the 2 km scale, and we assumed 500 m was wide enough to accommodate movement. A second corridor analysis following the simulated restoration shows the new corridor to the north (Figure 6C). The corridor has 9.4% less cumulative resistance than the original (1348 weighted km *vs.* 1489 weighted km), and its least-cost path is 44% shorter in un-weighted length. A post-restoration barrier analysis indicates that the highest improvement scores now fall along the new corridor (Figure 6D); restoring a second 1 km^2^ swath in this new corridor at the point indicated by the arrow would further reduce *LCD* by 50%.

## Discussion

Connectivity models have provided valuable guidance to conservation planning efforts, as well as predictions of movement, gene flow, and isolation important to landscape genetics and other fields concerned with movement ecology. Yet they have almost exclusively emphasized identifying features that facilitate, rather than impede, movement; this emphasis gives an incomplete picture of how landscape features affect connectivity, what connectivity management strategies might be appropriate, and the uncertainty underlying model predictions. We see considerable potential for barrier detection analyses to help practitioners overcome these limitations. In particular, the ability to identify restoration opportunities can provide valuable alternatives to traditional conservation efforts focused on existing movement corridors.

Our reanalysis of the Columbia Plateau data (Figure 6) illustrates these points, showing how detecting barriers can increase conservation options available to practitioners, improve understanding of analysis products, and result in more robust conservation plans. Without a barrier analysis, conservation planners would likely have focused on conserving land in or adjacent to the original least-cost corridor. Our analysis revealed numerous opportunities to improve this corridor, but also that restoration of a 1 km^2^ swath of cropland would create a new corridor with several desirable characteristics. Specifically, the new corridor has a lower least-cost distance, is shorter in length, and appears to have fewer pinch-points (narrow sections) than the original corridor—all desirable characteristics for corridor design [15], [32]. Moreover, if the two original corridors remain in place, the new, northern corridor adds redundancy to connections between the natural landscape blocks. This is important because organisms seldom follow a single optimal path [43], and because redundant connections help to ensure continued connectivity in the face of unpredictable environmental changes [15].

The analysis showed that connectivity conservation options need not be limited to a small portion of the landscape, opening up much more area for actions that could conserve or enhance connectivity and illustrating tradeoffs between different conservation strategies and target locations. Beyond the corridor quality differences cited above, we note that the original corridor runs along a narrow stretch of land bordering Banks Lake, sometimes traversing cliffs. The cliffs were assigned low resistance because the landscape integrity model used by the Working Group only quantified the degree to which pixels have been converted to human land uses. Practitioners, however, may consider cliffs to be impermeable for some species of conservation concern. The barrier analysis allows the user to quickly focus a more critical examination of corridor characteristics on areas influencing the results, and to identify options for alternative corridors that may better fit specific planning needs.

Similarly, the analysis underscored the potential sensitivity of corridor mapping to errors in GIS base data: our results show how the misclassification of a single agricultural field could have entirely altered the location of the original least-cost corridor shown in Figure 6A. The sensitivity of connectivity analysis results to landscape features at key locations has consequences for disciplines that depend on corridor maps (like conservation planning) and for disciplines that depend on connectivity measures (like landscape genetics). We discuss applicability of barrier detection methods to sensitivity analysis and error checking below.

Following the first barrier analysis and simulated restoration, a subsequent barrier analysis indicated that the restoration would open up further restoration opportunities of considerable value, one of which would cut *LCD* values by half. Thus, simulating restorations and re-running corridor and barrier analyses will likely improve final conservation and restoration plans.

Although we are aware of no other efforts to automate identification of terrestrial connectivity restoration opportunities, least-cost corridor analyses have been used to guide placement of crossing structures across roads to restore connectivity for wildlife. For example, Beier et al. [44] assigned a single, finite resistance value to all segments of a highway between two protected areas, regardless of whether a segment contained wildlife crossing structures. The least-cost corridor between the areas crossed the highway at the location where a crossing structure would result in the lowest ecological cost of travel. If highway crossing structures were not located in this corridor, Beier et al. [44] recommended specific structures at particular locations. This approach is useful, but does not quantify the improvement compared to existing conditions, does not identify restoration opportunities outside of least-cost corridors, and cannot be readily applied to barriers more complex than roads.

In addition to overcoming these limitations, our method is also amenable to highlighting barriers that affect multiple corridors, introducing the concept of barrier centrality. As shown in Figure 4, barriers can be mapped across all patch pairs, and the results summed. This identifies barriers with high network centrality, similar to analyses that identify corridors or pathways with high centrality [21], [22], [45], [46].

### Applications for error checking and sensitivity analyses

GIS land cover data used to develop resistance layers for connectivity analyses are typically based on satellite or aerial imagery and often suffer from high levels of classification error [34], [47]. Although our method relies on these same base data, it can help to prioritize error checking of the data by highlighting mapped features that strongly influence corridor locations. If a permeable feature is misclassified as impermeable and identified as a barrier, the misclassification could entirely alter a corridor's location. We recommend examining detected barriers, either by manually checking aerial imagery or conducting field surveys. Similarly, impermeable features misclassified as permeable that occur along least-cost paths can change corridor locations as well. Examining features along least-cost paths in tandem with barriers could thus further reduce the effects of classification error in connectivity analysis products.

Barrier detection can also be applied to parameter sensitivity analyses, important because resistances are often assigned based on expert opinion, which can be unreliable [34], [47], [48]. For example, if a given land cover type fell along a corridor's least-cost path or encompassed an influential barrier outside of the corridor, the resistance assigned to that land cover type would be known to influence the corridor's location. The sensitivity of the corridor's location to the resistance value assigned the land cover type could then be analyzed using alternative parameterization methods as described by Beier et al. [47]. As with connectivity models, our method will depend on the grain size of the resistance raster; to adequately resolve features that potentially impede movement, we recommend pixels no larger than ½ the width of barriers one is interested in detecting.

### Potential enhancements

#### Directionality of barrier effects

Our methods could be improved to more precisely pinpoint barriers. For example, elongated moving windows (search polygons) could perform better than circles to identify the best path for an improved corridor design. Measuring *ΔLCD* along elongate polygons placed at different angles, although more computationally complex than measuring across circles, would allow the attribution of directionality to barrier effects as well as adjustment of improvement scores at large search distances to reflect improvement achievable at smaller (nested) distances. New procedures to select the best orientation and width of such polygons could obviate the need to subjectively orient restoration polygons, like the 500 m×2 km polygon in our simulated restoration (Figure 6).

#### Restoration cost

We measured barrier strength by conservation improvement per meter restored because it was the simplest way to illustrate our approach. An alternative metric would be conservation improvement per restoration dollar; this would reflect, for example, that the cost per meter of a 10 m road crossing structure exceeds the cost per meter of a 50 m crossing structure, which in turn exceeds the cost per meter of restoring agricultural land. This enhancement would facilitate incorporation of connectivity restoration into return-on-investment analyses [49], [50], helping managers to balance improvement potential, corridor importance, costs, and risk of conversion or degradation when deciding which parts of a landscape should be conserved or restored. The disadvantage, or course, is that this metric would require more data to calculate.

#### Restoration efficacy

Different *R′* resistance values could be applied to different land cover types to reflect the fact that some barriers would be more permeable to movement following restoration than others. For example, a highway underpass installed to allow animal movement may still have considerable resistance, whereas a restored forest stand may have resistance similar to undisturbed forest.

#### Other enhancements

Just as areas that cannot be conserved can be removed from reserve selection algorithms [51], un-removable barriers, such as urban areas, could be excluded from barrier analyses. The metrics described in equation (2) could be modified to incorporate restoration costs that vary by feature type, or land prices mapped using parcel data. Metrics of corridor importance (e.g., link centrality) could be integrated by multiplying improvement scores by such metrics, which would highlight opportunities to restore the most potent barriers in the most important corridors. Or, rather than focusing on pairs of patches, the method could be altered to focus on the connectedness of each patch by summing barriers detected between each patch and all others. Lastly, improvement scores may be expressed in terms of absolute improvement or percent improvement relative to unrestored corridor resistance. An advantage of the latter approach is that it would favor restoration in corridors in which *LCD* values are already low, presumably meaning they are more viable.

Which of these enhancements are most valuable will depend on the objectives of individual users and projects.

### Application in other connectivity modeling frameworks

Although least-cost corridor models are by far the most commonly applied connectivity planning tool, they rely on simple assumptions about animal movement and other processes [43], [48], [52], [53]. However, our approach can be applied in any connectivity modeling framework that produces measures of effective distance. For example, circuit-based connectivity analyses can model the relative proximity of each pixel to two patches by setting the voltage of one patch to 1 and the other to ground (see [15] for details on applying circuit modeling to landscapes). The resulting voltage surface gives the probability that a random walker will reach one patch before reaching the other [15], [54]. Strong gradients in voltage indicate barriers that separate areas relatively accessible to one patch from areas relatively accessible to the other. If removed, such barriers would reduce effective resistance between the patches, an analog to *LCD* that takes into account the availability of multiple, parallel connections. A similar approach is widely used in microchip design: simulated voltage levels reveal areas with strong voltage gradients (known as IR drops) where electrical connectivity must be enhanced [55]. Thus barrier analysis using circuit theory can identify opportunities to provide valuable redundant connections even when *LCD* would not be reduced. In contrast, barrier analysis using least-cost methods will not identify these opportunities.

Individual-based movement models provide a more complex but also more powerful framework for modeling connectivity, capable of incorporating more biological realism and behavioral information than least-cost or circuit analyses [56]. As long as an individual-based model can produce maps of effective distance (e.g., based on the probability of, or energetic expenditure associated with, reaching different locations from a source patch), the approach described here could be applied to the model. Models such as PATH [16] and HexSim [17] can be used to derive such measures.

### Potential for integration with systematic conservation planning

Our method is not a substitute for algorithms like Marxan [57] or Zonation [51], which are designed to optimize selection of reserves or sets of conservation actions. Although our method identifies and ranks candidate areas for restoration actions, it does not select optimal *sets* or portfolios of conservation actions to achieve given conservation goals while minimizing cost. The same can be said for algorithms designed to map areas that most facilitate movement and connectivity (e.g., [22], [40], [46], [58]–[60]); rather than incorporating optimization routines, such algorithms instead produce maps that must be interpreted by practitioners, who then make conservation decisions in light of costs, benefits, and other management objectives.

Although it has long been recognized as important to reserve network design [61], incorporating connectivity directly into optimization algorithms has proven difficult. Most such efforts can be characterized as minimizing local fragmentation by either considering the geographic proximity of candidate areas to other areas (e.g., [62]–[64]) or maximizing the compactness and contiguity of reserves by favoring selection of adjacent cells or using boundary quality or length penalties (e.g., [29], [57], [65], [66]). Because these algorithms favor conserving or restoring contiguous natural areas, they may neglect areas that, although fragmented, contribute to connectivity between natural areas. Thus, relying solely on maximizing the proximity or contiguity of protected areas could lead to elimination of movement routes that cross human-dominated landscapes.

Progress toward synthesizing connectivity and optimization algorithms has likely been hampered by the ‘network’ nature of connectivity planning: conservation in one area can affect the function and value of distant areas, contingent upon the conservation status and characteristics of the intervening landscape. Incorporating this complexity into optimization algorithms becomes computationally prohibitive with large numbers of planning units [67]. Still, practitioners are beginning to use outputs of multi-species connectivity models as inputs to optimization algorithms like Zonation [23], [68]. Such examples are promising, and should be equally applicable with restoration-oriented algorithms such as ours.

An alternative to our approach that would seek to develop a near-optimal set of conservation actions would be to employ a routine similar to that used by Zonation software, which begins with an intact landscape and iteratively removes grid cells with low conservation value [51], [69]. Starting with a landscape in which all restorable barriers have been removed, different sets of barriers could be added back in and connectivity metrics recalculated at each iteration. As with traditional connectivity models, however, this would be computationally prohibitive with large numbers of patches or restoration sites because of the computational time required for recalculating connectivity metrics. A promising hybrid approach could be to use the method described in this paper to identify sets of pre-screened restoration opportunities, which could then be removed from a resistance surface and added back in using an algorithm like Zonation's.

### Practical considerations for improving conservation and restoration decisions

Managing for connectivity to facilitate gene flow, climate adaptation, and other processes is challenging without reliable maps to guide practitioners [33]. Connectivity analyses have provided valuable implementation guidance in the past; barrier mapping can increase the rigor of such analyses and the range of conservation options they reveal. It can help practitioners a) decide if connectivity conservation is a worthy investment in a landscape; b) identify opportunities to restore vs. conserve different areas; c) reduce uncertainty due to errors in GIS base data; and d) balance potential improvement against costs so that investments can be prioritized.

The goals of managers and planners can be used to guide applications of barrier detection methods. For example, if a transportation agency is interested in determining which highway segments are likely to have the greatest impact on wildlife movement, the search window should correspond to the width of highways, with outputs clipped to highways and the *R′* value determined based on the estimated resistance of the kind of crossing structure (or alternative structures) being considered. If a land management agency is prioritizing restoration of degraded native vegetation, the search window should relate to the size of appropriate restoration projects, and outputs should be clipped to the eligible land base (e.g., limited to the type of vegetation the restoration would target). If an NGO is identifying landowners interested in obtaining voluntary incentive payments for wildlife-friendly management, the window should reflect the scale of such management. Summarizing barrier analyses across multiple scales will be desirable for collaborations among organizations with differing goals and mandates. As noted above, iterative application of the model with simulated restorations will likely provide the most informative results and most robust conservation plans.

Similarly, the method may have potential to help adapt results from coarse-filter connectivity assessments, such as landscape integrity/human modification-based connectivity maps, to more fine-filter objectives (see [70] for a review of coarse- and fine-filter conservation planning). Alternative corridors revealed by the method could be assessed for their suitability under different planning constraints (e.g., corridors for species that must avoid cliffs, as in the Columbia Plateau example). While not a replacement for species-specific connectivity analyses, such an approach could help land managers evaluate alternatives if a mapped corridor is deemed unsuitable for their particular needs.

Connectivity maps do not always identify functioning routes that need to be maintained and protected; rather, they frequently map routes that may not be currently viable, but appear to provide the best opportunities for future work toward enhancing connectivity. In this sense connectivity maps often represent visions and goals for desired future conditions [71]. Barrier detection can add insight into the practicality of these goals, and identify specific options for achieving them. It can also help practitioners to ‘triage’ a connectivity plan, identifying corridors that traverse numerous barriers – and therefore would require significant investment to fully restore – so that efforts may be focused on more viable movement routes.

Perhaps most importantly, the ability to detect options to re-route corridors also opens up a broader suite of potential actions to improve connectivity. It can help managers identify new corridors that add additional movement pathways in areas important to the overall connectivity of a landscape (i.e. linkages with high centrality). Combined with spatially explicit land cost data, the method could help to improve conservation efficacy while reducing costs.

We hope barrier analyses will expand conservation options available to managers, and broaden conversations about restoration of connectivity more generally. By identifying new ways to improve connectivity in a particular area, the method can allow managers to consider different suites of strategies, or engage with new sets of stakeholders with interests in different areas. Both from the perspective of entities mandated to carry out conservation actions, and from the perspective of stakeholders with interests in the lands that are the focus of such actions, broadening the suite of alternatives and tools can only increase the opportunities for finding common ground in pursuit of multiple objectives.
